# GATA2 Deficiency Presenting With a WHIM‐Like Phenotype and Subsequent Development of Monosomy 7‐Associated Myelodysplastic Syndrome in an Adolescent Female: A Case Report

**DOI:** 10.1002/ccr3.73869

**Published:** 2026-11-03

**Authors:** Aziz Eghbali, Meshkat Poortavakol, Hamideh Sadat Mirmohammadi

**Affiliations:** ^1^ Clinical Research Development Center of Aliasghar Hospital Iran University of Medical Sciences Tehran Iran; ^2^ Department of Hematology and Blood Banking, School of Allied Medical Sciences Iran University of Medical Sciences Tehran Iran; ^3^ Department of Pediatrics, School of Medicine Iran University of Medical Sciences Tehran Iran

**Keywords:** GATA2 deficiency, immunodeficiency, monocytopenia, monosomy 7, myelodysplastic syndrome, WHIM syndrome

## Abstract

We report an 18‐year‐old female with GATA2 deficiency presenting with a WHIM‐like phenotype, including pancytopenia, monocytopenia, CD19^+^ B‐cell depletion, sensorineural hearing loss, and cutaneous warts. Subsequent cytogenetic evaluation revealed monosomy 7 and 7q22 deletion, with bone marrow progression to MDS. She was treated with azacitidine and referred for allogeneic HSCT.

## Key Clinical Message

GATA2 (GATA‐binding protein 2) deficiency can initially present with a WHIM‐like phenotype, including warts and monocytopenia; its diagnosis should be considered in adolescents with unexplained cytopenias and syndromic features. Early genetic testing enables timely surveillance for monosomy 7 and myelodysplastic syndrome (MDS), facilitating curative hematopoietic stem cell transplantation (HSCT) before leukemic transformation.

## 1 Introduction

GATA2 represents a zinc finger‐containing transcription factor that serves as a pivotal regulator of hematopoiesis, with essential functions in hematopoietic stem cell homeostasis and lymphocyte lineage development [1]. Individuals harboring mutations in GATA2 exhibit a broad spectrum of clinical abnormalities, including cytopenias affecting B‐cell, natural killer (NK)‐cell, lymphocyte, and monocyte subsets, heightened susceptibility to infections due to immunodeficiency, as well as pulmonary alveolar proteinosis and pulmonary hypertension [2, 3]. Moreover, the disorder may present with severe bone marrow failure (BMF), diverse immunodeficiency phenotypes, or syndromic congenital anomalies, or may due to incomplete or partial penetrance, remain entirely asymptomatic in a small proportion of mutation carriers [4, 5].

Although the presentation of GATA2 deficiency varies widely, the associated clinical features are now understood to constitute a unified monogenic entity. This syndrome was formally introduced as a distinct category of “myeloid neoplasms with germline GATA2 mutations” in the revised 2016 World Health Organization (WHO) classification of hematopoietic tumors. The spectrum of GATA2 deficiency includes several cellular immunodeficiency disorders such as MonoMac syndrome (characterized by monocytopenia and susceptibility to mycobacterial infections) [6, 7], Emberger syndrome (marked by myelodysplasia and lymphedema) [8], and DCML deficiency (affecting dendritic cells, monocytes, B‐lymphocytes, and NK‐lymphocytes) [9]. In addition, the syndrome encompasses myeloid malignancies, most notably familial and primary pediatric forms of MDS and acute myeloid leukemia (AML) [4, 10, 11, 12]; patients with GATA2 deficiency face a high predisposition to these conditions, with a median age at onset of 17 years [5, 13]. Germline mutations in GATA2 have further been linked to a range of other clinical findings, including chronic neutropenia [6], hypocellular BMF [14, 15], defects in megakaryopoiesis, B‐ and NK‐cell deficiency [16, 17], congenital auditory and neurological abnormalities (predominantly sensorineural deafness and attention‐deficit/hyperactivity disorder (ADHD)) [18, 19], as well as recurrent pregnancy loss [6, 18, 20].

Herein, we report an adolescent female with multisystem manifestations including sensorineural hearing loss, congenital heart disease, immunologic abnormalities, and persistent pancytopenia, who was ultimately diagnosed with GATA2 deficiency and subsequently developed monosomy 7‐associated MDS. This case highlights the diagnostic challenge of distinguishing GATA2 deficiency from warts, hypogammaglobulinemia, infections, and myelokathexis (WHIM) syndrome in the setting of monocytopenia and extensive cutaneous warts.

## 2 Case History

The patient, an 18‐year‐old female born to consanguineous parents, was delivered prematurely at 34 weeks of gestation with a birth weight of 1900 g. Her early childhood was marked by cochlear implantation at age 3 for sensorineural hearing loss and surgical repair of an atrial septal defect at age 4. At age 13, she presented to the endocrinology clinic with growth retardation (weight 33.7 kg and height 135.5 cm); laboratory evaluation revealed anemia (hemoglobin 9.1 g/dL), thrombocytopenia (platelets 130 × 10^9^/L) and leukopenia (white blood cell count 1 × 10^9^/L), accompanied by marked monocytopenia (0%), left‐shifted granulopoiesis, and toxic granulation on peripheral blood smear, leading to hematology referral.

Additional history included severe progressive vision impairment over the preceding month with a refractive error ranging from −7 to −9 diopters, numerous warts involving the lower extremities, and multiple oral aphthous ulcers, which had partially resolved at the time of evaluation. Physical examination demonstrated clear lung auscultation, no lymphadenopathy, a soft nontender abdomen, mild hepatomegaly (liver palpable 2 cm below the costal margin), and splenomegaly (spleen extending 6 cm below the costal margin). Abdominal ultrasonography confirmed splenomegaly (dimensions 52 × 135 mm) with normal echogenicity.

Follow‐up testing demonstrated persistent pancytopenia, with a hemoglobin level of 8.3 g/dL, white blood cell counts of 0.78 × 10^9^/L, and platelet count of 128 × 10^9^/L. Peripheral blood smear examination showed persistent monocytopenia (1%), anisocytosis (2+), giant platelets, and elevated RDW. A reticulocyte count of 3.24% was noted; however, the reticulocyte response was inappropriately low for the degree of anemia. Iron studies revealed low serum iron (< 10 μg/dL) and transferrin saturation (3%), consistent with impaired marrow production. A FANCA assay was reported as weakly positive. Bone marrow examination revealed a hypocellular marrow with erythroid hyperplasia and a myeloid‐to‐erythroid ratio of approximately 1:2. No morphologic evidence of MDS, increased blasts, or hematologic malignancy was identified at that time. Cytogenetic analysis was not performed during the initial evaluation at 13 years of age. Subsequent follow‐up was affected by family circumstances and financial limitations, which contributed to interruptions in the patient's medical follow‐up and delayed further diagnostic investigations.

## 3 Methods

An immunological evaluation was conducted using flow cytometry to assess lymphocyte subsets in peripheral blood, including CD3^+^, CD4^+^, CD8^+^, CD16^+^/56^+^ (NK cells), and CD19^+^ (B cells). The results indicated profound depletion of CD19^+^ B cells (< 1%) with relative T‐cell predominance. These findings provided significant context for the subsequent identification of a heterozygous GATA2 mutation.

Given the multisystem features including hearing loss, vision impairment, multiple lower‐extremity warts, anemia, pancytopenia, splenomegaly, bronchopneumonia, abdominal lipoma, immunodeficiency, and a history of atrial septal defect, WHIM syndrome was initially suspected. During this period, the patient was managed with monthly intravenous immunoglobulin (IVIG) therapy at a dose of 10 g and prophylactic antimicrobial therapy, including acyclovir and co‐trimoxazole because of the suspected underlying immunodeficiency.

Whole‐exome sequencing (WES) was performed at 16 years of age using DNA extracted from peripheral blood. WES identified a heterozygous likely pathogenic frameshift variant in GATA2 (Chr3:128200674 G>GT; NM_032638: ENST00000341105.2 Exon 5: c.1130dup: p.Tyr377*). The laboratory report classified this variant as likely pathogenic according to American College of Medical Genetics and Genomics (ACMG) criteria. The variant introduces a premature termination codon and is predicted to result in loss of GATA2 function. No pathogenic or likely pathogenic variants were identified in CXCR4 or CXCR2, making a molecular diagnosis of WHIM syndrome less likely (Table 1).

**TABLE 1:** Whole‐exome sequencing report obtained at 16 years of age, demonstrating a heterozygous likely pathogenic GATA2 c.1130dup (p.Tyr377*) variant and additional reportable variants. All variants were identified by whole‐exome sequencing performed on peripheral blood genomic DNA (Illumina NovaSeq 6000, 100 bp paired‐end, 100 × coverage, GRCh37 assembly, Agilent SureSelect Human All Exon V7). Variant allele frequencies were not reported in the laboratory report (see Methods). Complete variant nomenclature for the C7 variant was not provided in the original laboratory report.

Gene	Variant nomenclature	Variant type	Zygosity	ACMG classification
GATA2	Chr3:128200674 G>GT; NM_032638: ENST00000341105.2 Exon 5: c.1130dup: p.Tyr377*	Frameshift (duplication)	Het.	Likely pathogenic
FAM149B1	Chr10:74995003 ACTAT>A; NM_173348.2: ENST00000242505.6: Exon 12: c.1531_1534del: p.Tyr511Serfs*88	Frameshift (deletion)	Het.	Likely pathogenic
ACAD8	Chr11:134131195 GC>G; NM_014384.3: ENST00000281182.4: Exon 8: c.870del: p. Ser291Leufs*41	Frameshift (deletion)	Het.	Likely pathogenic
SERPINA1	Chr14:94849160 C>T; NM_000295.5: ENST00000393087.4: Exon 2: c.415G>A: p. Gly139Ser	Missense	Het.	Pathogenic, Others
EGF	Chr4:110909017 G>A; NM_001963.6: ENST00000265171.5: Exon 17: c.2608 + 1G>A	Splice site	Het.	Likely pathogenic
NPHP3	Chr3:132416201 C>T; NM_153240.5: ENST00000337331.5: Exon 14: c.1991G>A: p. Trp664*	Nonsense	Het.	Likely pathogenic
MYBPC3	Chr11:47357494 G>A; NM_000256.3: ENST00000545968.1: Exon 26: c.2671C>T: p. Arg891Trp	Missense	Het.	VUS
ING1	Chr13:111367793 G>A; NM_198219.3: ENST00000333219.7: Exon 1: c.136 + 1161G>A	Intronic	Het.	Likely pathogenic
DES	Chr2:220286189 A>G; NM_001927.4: ENST00000373960.3: Exon 6; c.1151A>G; p. His384Arg	Missense	Het.	Likely pathogenic
C7	Chr5:40959622 C>A;	Not specified	Het.	Likely pathogenic

Abbreviations: Het., heterozygote; VUS, variant of uncertain significance.

The WES report also identified heterozygous variants in FAM149B1, ACAD8, SERPINA1, EGF, NPHP3, MYBPC3, ING1, DES, and C7. These variants were reported by the testing laboratory as reportable variants according to ACMG criteria and as variants reported as pathogenic or likely pathogenic in the ClinVar database, but were considered unrelated to the patient's presenting phenotype in the laboratory report. Their detailed nomenclature, variant type, zygosity, and laboratory classifications are summarized in Table 1.

Variant allele frequencies (VAFs) were not provided in the available laboratory report, and no non‐hematopoietic tissue was available for independent confirmation of germline status. Therefore, although the clinical phenotype strongly supports GATA2 deficiency, the germline origin of the identified GATA2 variant could not be independently confirmed by testing an alternative non‐hematopoietic tissue. Likewise, the available data do not permit definitive assignment of the additional variants as germline or somatic. These limitations were considered when interpreting the genetic findings.

## 4 Conclusions and Results

Subsequent cytogenetic evaluation at age 18 involved karyotyping. Twenty metaphase spreads were studied using GTG‐banding at 400–450 band resolution. Cytogenetic analysis demonstrated monosomy 7 in all examined abnormal metaphases. Two metaphases showed isolated monosomy 7, whereas the remaining 18 metaphases harbored monosomy 7 together with an additional marker chromosome (Figure 1). Fluorescence in situ hybridization (FISH) using an MDS probe panel revealed a 7q22 (RELN) deletion in 87 of 125 interphase nuclei (71%), with no abnormalities detected by the remaining probes (SEMA5A, EGR1, D7Z1, D8Z1, PTPRT) (Figure 2). Furthermore, bone marrow flow cytometric analysis demonstrated a distribution of approximately 40% mature lymphoid cells, 40% mature myeloid cells, and 20% monocytes. Consistent with the earlier peripheral blood immunophenotyping findings, CD19^+^ B cells remained below 1%. Morphologic evaluation showed pancytopenia with dysplastic changes involving both erythroid and myeloid lineages, confirming the diagnosis of MDS. Following the diagnosis of MDS, azacitidine therapy was initiated. Azacitidine was administered at a dose of 100 mg daily for five consecutive days in monthly cycles. The treatment was given as disease‐directed therapy for MDS while the patient is currently awaiting allogeneic hematopoietic stem cell transplantation. The patient remained under ongoing azacitidine treatment, and formal assessment of hematologic response was pending completion of further treatment. A summary of the clinical timeline, including key investigations and treatments, is provided in Table 2.

**FIGURE 1:**
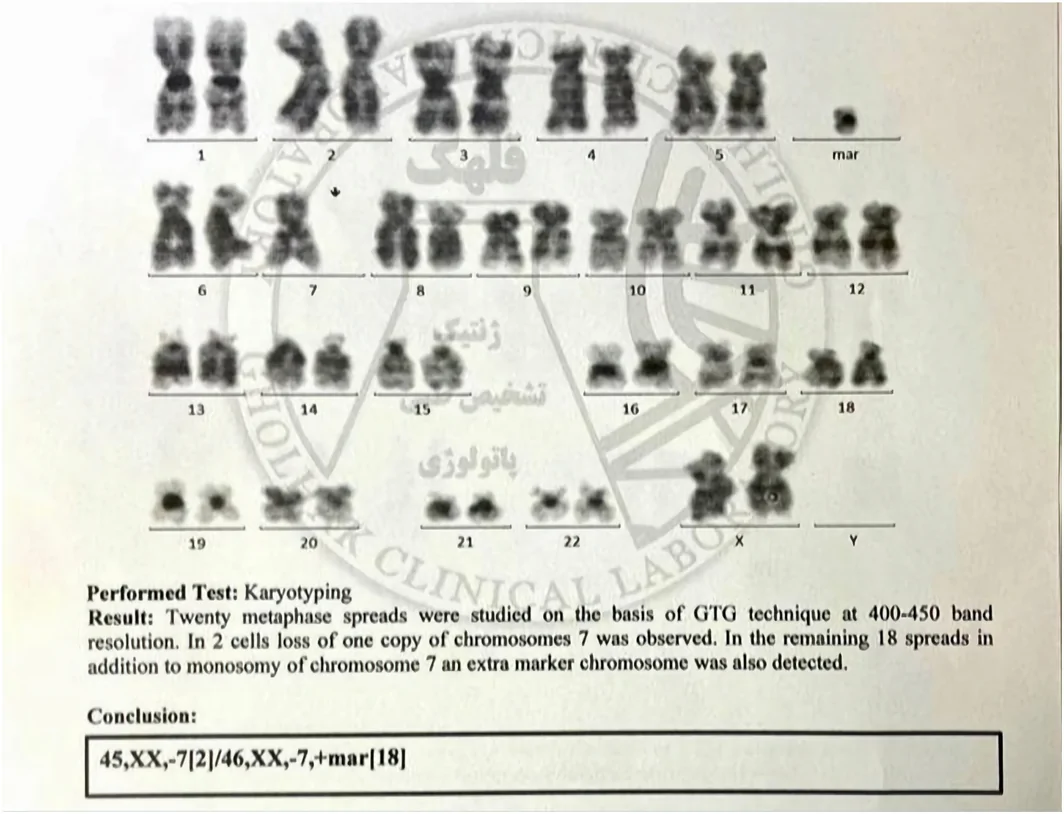
GTG‐banded karyotype obtained at age 18, demonstrating monosomy 7 and an additional marker chromosome.

**FIGURE 2:**
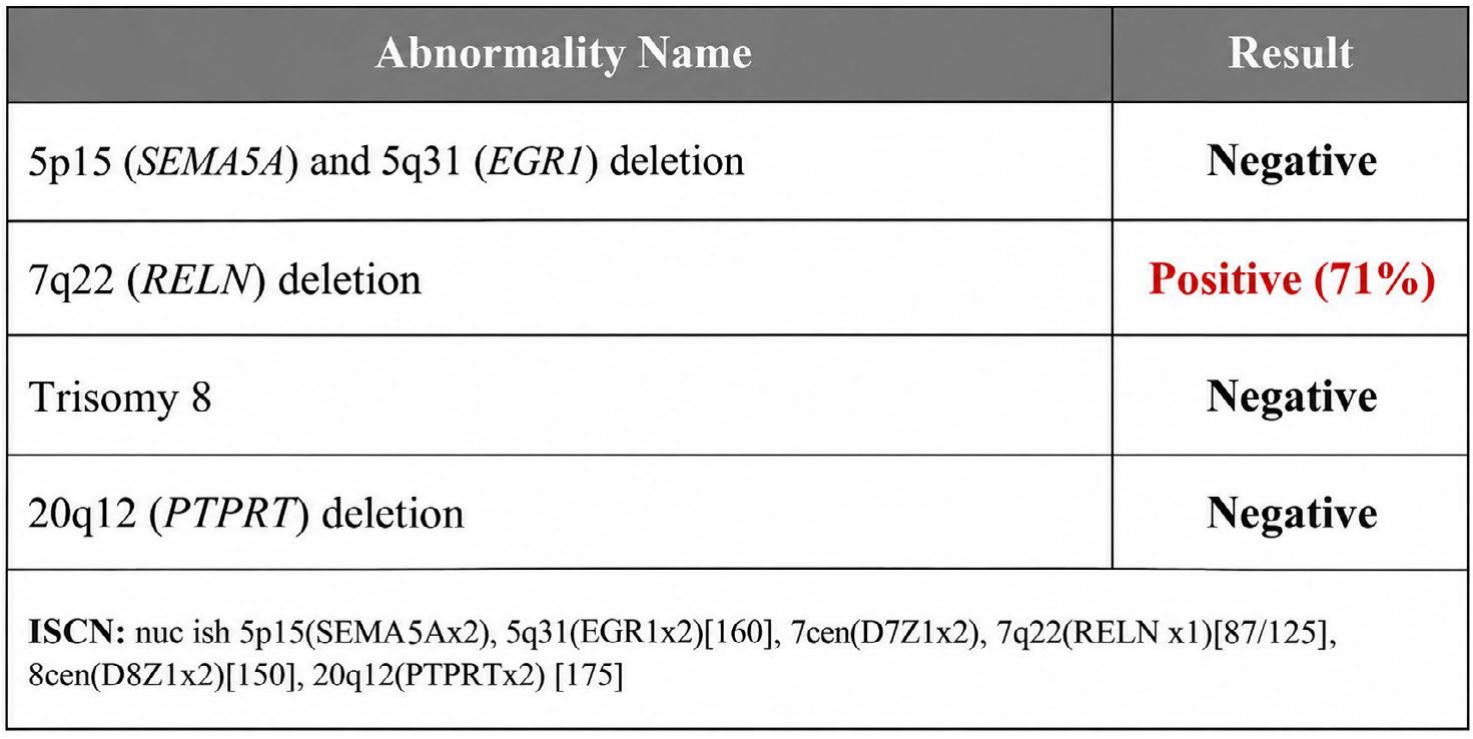
FISH analysis using an MDS probe panel, demonstrating deletion of 7q22 (RELN) in a significant proportion of interphase nuclei.

**TABLE 2:** Timeline of clinical presentation, investigations, and treatment.

Age	Clinical event	Investigation/treatment
Birth	Premature birth (34 weeks, 1900 g)	—
3 years	Sensorineural hearing loss	Cochlear implantation
4 years	Atrial septal defect	Surgical repair
13 years	Growth retardation, pancytopenia, profound monocytopenia, cutaneous warts, aphthous ulcers, hepatosplenomegaly, B‐cell deficiency	Bone marrow examination showed hypocellularity and erythroid hyperplasia without morphologic MDS or increased blasts. Monthly IVIG (10 g) and antimicrobial prophylaxis were initiated.
13–16 years	Persistent hematologic and syndromic abnormalities	Follow‐up was delayed because of family circumstances and financial limitations, contributing to delayed further diagnostic investigations.
16 years	Persistent cytopenias and immunologic abnormalities	WES using peripheral blood DNA identified a heterozygous likely pathogenic GATA2 c.1130dup; p.Tyr377* variant; additional variants were also detected
18 years	MDS with monosomy 7 diagnosed	Karyotype demonstrated monosomy 7 with an additional marker chromosome; FISH demonstrated 7q22 (RELN) deletion in 87/125 nuclei (71%) Azacitidine 100 mg/day for 5 consecutive days in monthly cycles; referred for allogeneic HSCT and awaiting transplantation

## 5 Discussion

Autosomal dominant or de novo loss‐of‐function mutations in the GATA2 gene give rise to GATA2 deficiency, a disorder defined by impaired hematopoietic, immune, and lymphatic function [4, 21]. Although rare, GATA2 deficiency produces a diverse spectrum of clinical involvement affecting hematopoietic, immune, lymphatic, pulmonary, dermatologic, and neurologic systems. Beyond the classic phenotypes—Emberger syndrome, MonoMAC syndrome, and DCML deficiency—other less common presentations have been documented, including pulmonary alveolar proteinosis (PAP) [21] and neurological abnormalities, and urogenital malformations have also been reported [13]. The pathogenic mechanisms underlying several manifestations of GATA2 deficiency, including hematopoietic dysfunction [22, 23], lymphedema [24, 25] and sensorineural hearing loss [26], have been investigated through laboratory and animal models. Our patient exhibited a striking constellation of these manifestations, including multiple lower‐extremity warts, cochlear implantation for sensorineural hearing loss, surgical repair of an atrial septal defect, persistent pancytopenia with profound monocytopenia, marked CD19^+^ B‐cell depletion, and hepatosplenomegaly.

A distinctive aspect of our case was the initial diagnostic consideration of WHIM syndrome, given the extensive cutaneous warts, profound monocytopenia, recurrent oral aphthous ulcers, and pancytopenia. However, WES identified a heterozygous GATA2 variant (c.1130dup; p.Tyr377*) with no pathogenic variants in CXCR4 or CXCR2, making WHIM syndrome highly unlikely.

A particularly notable finding in our patient was the persistent and profound depletion of CD19^+^ B cells (< 1%) observed in both peripheral blood and bone marrow immunophenotyping, a feature highly consistent with the immunologic phenotype reported in GATA2 deficiency. This finding occurred in conjunction with marked monocytopenia, another characteristic immunohematologic feature frequently observed in GATA2‐deficient patients.

GATA2 is critically involved in early embryonic hematopoietic specification through the endothelial‐to‐hematopoietic transition (EHT) [27, 28, 29]. Postnatally, GATA2 is highly expressed in HSCs and early hematopoietic progenitors [28, 29, 30, 31, 32], promoting myeloid‐erythroid progenitor differentiation [33, 34, 35, 36]. Maintaining HSC survival, self‐renewal, and differentiation in adult hematopoiesis requires GATA2 to interact with a variety of transcriptional regulators [37]. In GATA2 deficiency, interruption of these processes significantly increases the risk of hematopoietic dysfunction and confers a strong predisposition to MDS and AML [12].

GATA2 variants are classified into three functional categories: those altering protein structure (null variants including frameshift, truncating, nonsense, silent deleterious, splice‐region, and whole‐exon or whole‐gene deletion variants); those impairing zinc finger 2 (ZF2) domain functionality (missense mutations within ZF2); and those modulating allelic expression (variants located in the +9.5 kb intron 4 [32, 38] or −110 kb [39, 40] regulatory regions) [34, 41]. The first two categories induce GATA2 haploinsufficiency through complete or near‐complete loss of protein function, whereas regulatory region variants typically exert a hypomorphic effect [20, 39]. The identified variant (c.1130dup; p.Tyr377*) is a frameshift mutation introducing a premature termination codon, predicted to result in nonsense‐mediated mRNA decay and consequent loss of function.

Patients with GATA2 deficiency often exhibit a range of recurrent cytogenetic aberrations and somatic mutations across numerous genes, which frequently accompany the progression to myeloid malignancies. The most frequently encountered clonal cytogenetic abnormality in this setting is monosomy 7, reported in close to half of all GATA2‐deficient individuals who develop myeloid neoplasms [13]. When present alongside a 7q17 deletion or the unbalanced translocation der(1;7)(q10;p10) [4, 14, 42], monosomy 7 consistently produces monoallelic loss of 7q, a genetic feature that confers a poor prognosis [41, 43]. Focusing on pediatric MDS with monosomy 7, germline GATA2 mutations are identifiable in 37% of affected children. This percentage increases dramatically in the adolescent subgroup, in whom GATA2 deficiency underlies 72% of monosomy 7 cases [43, 44]. As shown in Figure 1, karyotyping revealed monosomy 7 with an additional marker chromosome, while FISH analysis (Figure 2) confirmed a 7q22 deletion in 71% of interphase nuclei.

Beyond the GATA2 variant, WES identified nine additional heterozygous variants classified per ACMG SF v3.0 secondary findings criteria (Table 1). Four, including FAM149B1 (Joubert syndrome 36), ACAD8 (isobutyryl‐CoA dehydrogenase deficiency), EGF (renal hypomagnesemia 4), and NPHP3 (Meckel syndrome 7, nephronophthisis 3, renal‐hepatic‐pancreatic dysplasia 1), follow autosomal recessive inheritance; as the patient carries only a single heterozygous allele at each locus and shows none of the associated phenotypic features, these findings most likely represent asymptomatic carrier status rather than a contributory diagnosis. The C7 variant, for which an inheritance pattern was not specified in the laboratory report, is associated with C7 deficiency, a complement pathway disorder; heterozygous carriers of complement component deficiencies are typically asymptomatic, consistent with the absence of a complement‐deficiency phenotype (e.g., recurrent Neisseria infections) in this patient.

The remaining four variants warrant individual comment. The SERPINA1 variant, reported as “Pathogenic, others” by the laboratory and linked to AAT deficiency‐related emphysema/cirrhosis and antithrombin Pittsburgh, has not been accompanied by a pulmonary or hepatic phenotype to date; its clinical significance in the heterozygous state remains uncertain. The MYBPC3 variant, a VUS associated with autosomal dominant cardiomyopathies, has shown no echocardiographic correlate beyond the previously repaired atrial septal defect, though its AD inheritance pattern may warrant cardiology surveillance. Similarly, the DES variant, linked to AD dilated cardiomyopathy and myofibrillar myopathy, has not manifested a myopathic or cardiac phenotype but may merit longitudinal follow‐up. The ING1 variant, reported in association with somatic (not germline) head and neck squamous cell carcinoma, has unclear relevance to a constitutional finding, and its significance cannot be determined from available evidence.

Collectively, none of these nine variants provides an alternative or contributory explanation for the patient's hematologic and immunologic phenotype, which remains most parsimoniously explained by the likely pathogenic GATA2 variant. The AD‐associated cardiomyopathy variants and the AR carrier findings may nonetheless be relevant for future surveillance and family counseling, which has been recommended accordingly.

Another notable feature of this case is the prolonged interval between the initial presentation with cytopenias and syndromic manifestations and the eventual diagnosis of GATA2 deficiency with clonal evolution. The patient initially demonstrated hypocellular marrow, persistent pancytopenia, profound monocytopenia, and B‐cell deficiency without definitive morphologic evidence of MDS. Over time, cytogenetic abnormalities, including monosomy 7 and 7q22 deletion emerged, followed by progression to MDS. This clinical course illustrates the dynamic nature of GATA2 deficiency and emphasizes the importance of repeated hematologic, cytogenetic, and molecular evaluation in patients with unexplained cytopenias, even when initial marrow findings are nondiagnostic.

To date, allogeneic HSCT constitutes the sole curative intervention available for GATA2 deficiency. For individuals deemed high‐risk, there is broad agreement that the most favorable window for preemptive HSCT falls within the hypocellular phase of MDS, before the emergence of serious sequelae such as opportunistic infections or transformation to MDS‐EB or AML [5, 10, 41, 45, 46, 47]. Our patient was subsequently referred for allogeneic HSCT and is currently awaiting transplantation.

## Author Contributions

**Aziz Eghbali:** supervision, validation, project administration, conceptualization, investigation, methodology, writing – review and editing. **Meshkat Poortavakol:** writing – review and editing, writing – original draft, formal analysis, data curation, methodology, visualization. **Hamideh Sadat Mirmohammadi:** project administration, validation, supervision, investigation, writing – review and editing, methodology.

## Funding

The authors have nothing to report.

## Consent

Written informed consent was obtained from the patient for publication of this case report and any accompanying images. A copy of the signed consent form is retained by the corresponding author and will be provided to the Editor upon request.

## Data Availability

The data that support the findings of this study are available on request from the corresponding author. The data are not publicly available due to privacy or ethical restrictions.
